# Novel Therapeutic Targeting of CCL3-CCR4 Axis Mediated Apoptotic Intesitnal Injury in Necrotizing Enterocolitis

**DOI:** 10.3389/fimmu.2022.859398

**Published:** 2022-04-22

**Authors:** Xi Yuan, Zihan Xiong, Wei Liu, Yue Li, Hongdong Li, Xuemei Zhang, Yibing Yin, Pingyong Xu, Ju Cao, Dapeng Chen, Zhixin Song

**Affiliations:** ^1^Department of Clinical Laboratory, National Clinical Research Center for Child Health and Disorders, Ministry of Education Key Laboratory of Child Development and Disorders, Chongqing Key Laboratory of Child Infection and Immunity, Children’s Hospital of Chongqing Medical University, Chongqing, China; ^2^Department of Gastrointestinal and Neonatal Surgery, Children’s Hospital of Chongqing Medical University, Chongqing, China; ^3^Molecular Medicine and Cancer Research Center, College of Basic Medical Sciences, Chongqing Medical University, Chongqing, China; ^4^Department of Emergency Medicine, Children’s Hospital of Chongqing Medical University, Chongqing, China; ^5^Department of Laboratory Medicine, Key Laboratory of Diagnostic Medicine, Chongqing Medical University, Chongqing, China; ^6^Key Laboratory of RNA Biology, National Laboratory of Biomacromolecules, Chinese Academy of Sciences (CAS) Center for Excellence in Biomacromolecules, Institute of Biophysics, Chinese Academy of Sciences, Beijing, China; ^7^College of Life Sciences, University of Chinese Academy of Sciences, Beijing, China; ^8^Department of Laboratory Medicine, The First Affiliated Hospital of Chongqing Medical University, Chongqing, China

**Keywords:** NEC, CCL3, CCR4, apoptosis, immunotherapy

## Abstract

**Background:**

Necrotizing enterocolitis (NEC) is the leading cause of neonatal gastrointestinal-related death, while the etiology and pathogenesis are poorly understood.

**Methods:**

The levels of CCL3 in intestinal tissue from modeling mice and patients were measured and analyzed. HE staining, TUNEL, Annexin and FCM were used to assess pathological changes and apoptosis in intestinal tissue and epithelial cells. CCL3, CCR4, cytokines, tight junction protein ZO-1, apoptosis-related genes and ERK1/2-NF-κB signaling pathway were detected by ELISA, Q-PCR, Western blotting and immunofluorescence.

**Results:**

CCL3 levels in the intestinal tissue significantly elevated in patients with NEC and mouse models. Blockade of CCL3 significantly alleviated NEC-related intestinal tissue damage, while administration of recombinant CCL3 aggravated intestinal injury by exacerbating intestinal epithelial cell apoptosis in NEC mice. Importantly, CCR4 blockade reversed CCL3-mediated damage to intestinal tissue and intestinal epithelial cell apoptosis both *in vivo* and *in vitro*. Further mechanistic studies showed that CCL3 regulated apoptosis-related BAX/BCL-2 expression through the activation of the ERK1/2 and NF-κB pathways, which could be reversed by anti-CCR4 treatment. Furthermore, ERK1/2 inhibition reduced CCL3-mediated phosphorylation of NF-κB in IEC-6 cells, while inhibition of NF-κB had no obvious effect on ERK1/2 phosphorylation. As expected, inhibition of NF-κB regulated BAX/BCL-2 expression and alleviated CCL3-induced epithelial cell apoptosis. These results indicate that high expression of CCL3 in NEC lesions promotes intestinal epithelial apoptosis through the CCL3-CCR4-ERK1/2-NFκB-BAX/BCL2 signalling axis, thereby exacerbating NEC-related intestinal injury.

**Conclusions:**

Our study represents an important conceptual advance that CCL3 may be one of the key culprits of intestinal tissue damage in NEC patients, and blocking either CCL3, CCR4, or NF-κB may represent a novel effective immunotherapy for NEC.

## Introduction

Necrotizing enterocolitis (NEC) is one of the most common and devastating gastrointestinal emergency during the neonatal period (1). As a critical condition primarily occurring in premature and low-birthweight infants (2), NEC endangers the lives of more than 15 million premature infants annually, particularly those with gestation ≤33 weeks or birth weight ≤2500g, with morbidity and mortality rates of up to 13% and 20–30%, respectively (3, 4). Moreover, children who undergo surgery for intestinal perforation have mortality rates as high as 50% (5). NEC is characterized by intestinal damage, ranging from mucosal injury to full-thickness necrosis and perforation, and is frequently accompanied by various complications, including intestinal fistula, short bowel syndrome, growth retardation, and neurodevelopmental disorders (2). However, the pathogenesis of NEC remains highly controversial. Therefore, a better understanding of its pathogenesis is of great significance for improving NEC treatment.

The cellular inflammatory environment of NEC is characterized by the activation of cytokine production and the infiltration and activation of neutrophils, macrophages, and lymphocytes, all of which together constitute the initiation and driver of NEC progression (6, 7). Multiple cytokines and chemokines [e.g., CCL2 (8), CCL5 (9), and CXCL8 (10)] derived from activated inflammatory cells play key roles in various inflammatory and infectious diseases. For example, some certain chemokines play important roles in the recruitment and activation of inflammatory cells in the inflammatory intestinal tissue of NEC (11, 12).

CC motif chemokine ligand 3 (CCL3), also known as macrophage inflammatory protein 1α (MIP-1α), is a pro-inflammatory chemokine which plays an important role in inflammation (13). CCL3, as a member of the CC chemokine family, can be secreted by various mature haematopoietic cells, including monocytes, macrophages, and neutrophils (14–16) under the stimulation of lipopolysaccharides (LPS) and other pro-inflammatory inducers during inflammation (17). CCL3 further binds to G protein-coupled receptors CCR1, CCR4, and CCR3 on immune cells (18), thus participating in the regulation of inflammation. Accumulating evidence has demonstrated that CCL3 is associated with the progression of multiple inflammatory/autoimmune diseases, including esophageal squamous cell carcinoma (19), chronic myeloid leukemia (20), and acute and chronic (fibrotic) liver damage (21), however, the effects of CCL3 on the regulation of inflammation, apoptosis, injury progression, and functional recovery in NEC remain unclear. In this study, our results uncovered a previously unrecognized role of CCL3 in the NEC progress, as CCL3 binds to CCR4 to mediate intestinal epithelial cell apoptosis, thereby exacerbating NEC progression. Therefore, blockade therapy targeting the CCL3-CCR4 axis may provide novel insights into NEC treatment.

## Methods

### Patients and Clinical Specimens

Modified Bell staging criteria (22, 23) were used to determine the severity of NEC. Neonates with severe NEC (Bell stage II or III) were enrolled in this study. Blood samples from patients at Bell stage II–III were collected to detect CCL3 expression, and blood samples from non-NEC preterm infants were used as controls. The gestational age, day age, delivery mode, feeding practices, and medical conditions of each infant in the control group were matched with those in the NEC group. Blood collection was performed within 24 h of NEC diagnosis in the NEC and control groups according to the above matching principle. Serum was isolated and frozen at -80°C for subsequent ELISA measurements. The intestinal tissue used in this study was the necrotic intestinal tissue that was surgically resected, and the control intestinal tissue was the non-necrotic edge adjacent to the necrotic site. The study was approved by the Ethics Committee of the Children’s Hospital of Chongqing Medical University and conducted in accordance with the principles of the Declaration of Helsinki. Informed consent was obtained from the parents or other custodians of all neonates in the study.

### Experimental NEC Model

This study was reviewed and approved by the Ethics Committee of Chongqing Medical University. Male or female neonatal C57BL/6 mouse pups (7–10 days old) were purchased from the Animal Center of Chongqing Medical University and maintained in a neonatal incubator at 28°C. NEC was induced as described (24) by gavage with 20–30 µL/g hypertonic formula milk 5 times daily, hypoxic treatment for 2 min (5% oxygen, 95% nitrogen), and cold asphyxia stress (4°C, 5 minutes) twice daily for 4 days. LPS (Sigma-Aldrich, MO, USA) was fed separately at 5 µg/g mouse body weight daily. The formula milk contained Similac 60/40 (Abbott Laboratories, Saint-Laurent, Canada) and Esbilac (PetAg, Hampshire, IL, USA). In the NEC+recombinant CCL3 (rCCL3) group, NEC was induced according to the above-mentioned protocol and the pups were injected with 20μL (50 ng/mL) of recombinant Mouse CCL3/MIP-1 alpha Protein (R&D Systems, MN, USA). In the NEC+ CCL3 neutralizing antibody (anti-CCL3) group, NEC was induced and the pups were intraperitoneally injected with 20 μL (50 ng/mL) of Mouse CCL3/MIP-1 alpha Antibody (R&D Systems). In the NEC+anti-CCR4 group, the pups were intraperitoneally injected with 20 μL (50 ng/mL) of CCR4 antibody KH-4F5 (Santa Cruz Biotechnology, TX, USA). In the NEC+PDTC group, the modeled mice were intraperitoneally injected with pyrrolidinedithiocarbamate ammonium (PDTC; S3633, Selleck Chemicals, TX, USA) 60 mg/kg. In the NEC control group, the pups were injected intraperitoneally with phosphate-buffered saline (PBS). Pups in the healthy control group were breastfed by their mothers without any stress factors. Pups were excluded because of improper feeding or death within 24 hours after NEC induction. all surviving pups were euthanized 96 h after NEC induction and ileum samples were collected for further analysis.

### Cell Culture

Intestinal epithelioid cell line 6 (IEC-6) was purchased from the American Type Culture Collection (ATCC, USA). The cells were placed in disposable cell culture plates and cultured in Dulbecco’s Modified Eagle’s medium (Gibco BRL, TX, USA) supplemented with 10% fetal bovine serum (Gibco BRL) and 1% penicillin/streptomycin (Gibco BRL) in cell culture incubators at 37°C and 5% CO2. Thereafter, the medium was refreshed every 48–72 h.

### Haematoxylin and Eosin (H&E) Staining

The ileum tissue was fixed with 4% paraformaldehyde for 24 h, dehydrated with a concentration gradient of ethanol, embedded in paraffin, cut into 5 μm sections, and stained with H&E. Blind histological assessment was used to assess the severity of the intestinal injury, and NEC was considered when the severity score was ≥2. The methods and details of the histological assessment are shown in **Table 1**.

**Table 1:** Evaluation scale of intestinal tissue injury.

Score	Histological change
0	The villi of intestinal mucosa were intact and normal in structure
1	Mild edema of villi, epithelial abscission was limited to the top of the villi
2	Villi are moderately damaged and necrotic
3	Villi are missing and crypts are still recognizable
4	Complete loss of mucosal epithelial structure or transmural necrosis

### ELISA Assay

CCL3 expression in mice and patients with NEC were assayed using ELISA kits (R&D Systems) according to the manufacturer’s instructions. The levels of IL-6 (BioLegend, CA, USA) and TNF-α (BioLegend) were measured suing ELISA kits according to the manufacturer’s instructions.

### Real-Time PCR (RT-PCR) Analysis

Total RNA was extracted from the whole intestinal tissue using TRIzol reagent (Invitrogen, MA, USA) and then reverse transcribed into cDNA using the PrimeScript RT Kit (RR037A, Takara, Shiga, Japan) according to the manufacturer’s instructions. RT-PCR was performed using the SYBR Premix Ex Taq II (Tli RNase H Plus) Kit (RR820A, Takara) and an Applied Biosystems 7500 Fast Real-Time PCR System (Applied Biosystems, CA, USA). All primer sequences were synthesized by Sangon Biotech (Shanghai, China). GAPDH was used to normalize all other genes, and relative expression was calculated using the 2^-ΔΔCT^ method. The gene-specific oligonucleotide primers used for RT-PCR are listed in **Table 1** (**Supplementary Files**).

### Western Blotting

Intestinal tissue was homogenized and added to RIPA lysate for protein extraction, centrifuged at 12,000 g for 10 min, the supernatant was collected, and SDS polyacrylamide gel electrophoresis was performed, and the protein was transferred to a PVDF membrane. After sealing in 5% skim milk for 2 h at 20-25°C, the corresponding antibody was added, and the membrane containing the primary antibody was incubated at 4°C overnight. Next, the membrane was washed three times with TBST, horseradish peroxidase-labeled goat anti-rabbit IgG antibody (1:8000) and goat anti-mouse IgG antibody (1:10000) were added and incubated at room temperature for 1 h, and then the membrane was washed three times with TBST. The ultra-sensitive ECL chemiluminescence kit was used to develop the PVDF membrane after exposure, and use Image J (National Institutes of Health, MD, USA) was used for quantification. The following antibodies were used in the test: anti-BAX (Abcam, Cambridge, UK), anti-BCL-2 (Abcam), anti-CCR4 (Santa Cruz Biotechnology), anti-p-Erk1/2 (Cell Signaling Technology [CST], MA, USA), anti-Erk1/2 (CST), anti-p-NF-κB (CST), anti-NF-κB (CST), anti-p-Akt (CST), anti-Akt (CST), anti-p-JAK (CST), and anti-JAK (CST).

The following antibodies were applied: anti-BAX (ab32503) and anti-BCL-2 (ab182858) from Abcam, and anti-CCR4 (sc-101375) from Santa Cruz Biotechnology.

### TUNEL Staining (Paraffin Sections)

Apoptosis in sections of ileal samples was assessed using a commercially available TUNEL staining kit (Roche Diagnostics, Basel, Switzerland), according to the manufacturer’s instructions.

### Immunohistochemistry (Paraffin Sections)

Paraffin sections were prepared in the same manner as that for H&E staining. Antigen retrieval was performed according to the primary antibody instructions. An appropriate amount of endogenous peroxidase blocker was added to the sample, which was incubated at room temperature for 10 min and then rinsed with PBS. Goat anti-mouse CCL3 antibody (1:40, AF-450-SP) was added to the specimen and incubated at 37°C for 60 min. The tissue was stained using the Anti-Goat HRP-DAB Cell & Tissue Staining Kit (brown; R&D Systems, CTS008) and counterstained with hematoxylin (blue).

### Immunofluorescence (Paraffin Sections)

The paraffin sections were dewaxed and placed in a repair box of EDTA antigen retrieval buffer (PH8.0) for antigen retrieval in a microwave oven, and 3% BSA was used for blocking for 30 min, after which the blocking solution was discarded. Slides were placed in a wet box and incubated with the corresponding primary antibodies, including anti-CCL3 (1:40, AF-450-SP, R&D Systems), anti-CCR4 (1:200, sc-101375, Santa Cruz Biotechnology), and anti-E-cadherin (1:200, 14472S, CST) at 4°C overnight. The slides were then incubated with the secondary antibodies CY3(G1223, Servicebio, Wuhan, China), FITC (G1222, Servicebio), and CY5(GB27303, Servicebio), and incubated at room temperature for 50 min in the dark. The slides were then incubated with DAPI solution at room temperature for 10 min to stain the nuclei. The fluorescent images were observed and preserved using a fluorescence microscope.

### Annexin V for Flow Cytometric Detection of Apoptosis

IEC-6 cells were pre-incubated overnight at a density of 1 × 10^5 cells/mL. After 24 h, the cells were pre-treated with rCCL3 (50 ng/mL), anti-CCL3 (50 ng/ml), and PDTC (200 μM) for 24 h and then stimulated with 100 μg/mL LPS for 4 h. Next, the cells were resuspended at 1 × 10^6 cells/mL, and then 5μl of annexin V-FITC and propidium iodide (Becton Dickinson and Company, NJ, USA) were added. Cell apoptosis was detected by flow cytometry (FCM) (Becton Dickinson and Company).

### Statistical Analysis

GraphPad Prism 7 (GraphPad Software, CA, USA) was used for all statistical analyzes and figures. Continuous data was expressed as mean ± SD, and ordinal data was presented using median and interquartile range. Mann–Whitney U test or one-way ANOVA was performed for comparisons between two or more groups, and log-rank test with Kaplan-Meier analysis was conducted for survival. Statistical significance was set at p< 0.05.

## Results

### CCL3 Is Upregulated in Intestinal Necrotic Lesions of Patients With NEC and a Murine Model of NEC

**Figure 1:**
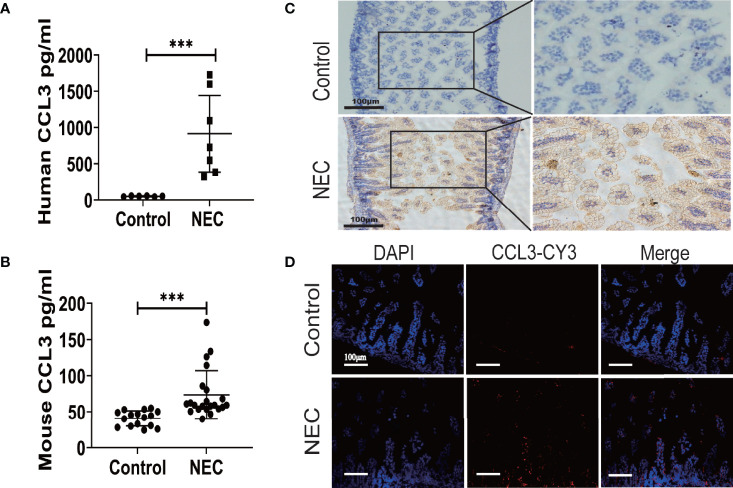
The Expression of CCL3 in the intestine necrotic lesions of patient and mice. ELISA was used to detect the concentration of CCL3 in intestinal tissue lysates from **(A)**. clinical patients and **(B)**. model mice and their corresponding control groups. Data are expressed as mean ± SD and were analyzed using the nonparametric Mann-Whitney U test. ***p < 0.001 versus Control or NEC groups. **(C)**. Immunohistochemistry was used to detect CCL3 in paraffin sections of mouse intestinal tissue (DAB chromogenic, Scale bar=100μm). **(D)** Immunofluorescence was used to detect CCL3 (CY3-Tyramide, red) in paraffin sections of mouse intestinal tissue as indicated (Scale bar=100 um).

To determine CCL3 expression in NEC, ELISA was used to detect CCL3 levels in intestinal tissue homogenates from clinical NEC patients and mice. The results showed that CCL3 expression in the necrotic intestine was significantly higher in patients with NEC than in non-necrotic intestines (**Figure 1A**). Similarly, CCL3 levels in the intestinal tissues of NEC model mice were significantly higher than those of control mice (**Figure 1B**), which was further verified by immunofluorescence (IF) and immunohistochemistry (IHC) staining, revealing that CCL3 expression in the intestinal tract of NEC mice was significantly higher than that in the control group, predominantly in the epithelium and lamina propria of the intestinal mucosa (**Figures 1C, D**). The co-occurrence of NEC with increased CCL3 concentration suggests that CCL3 may play a role in NEC pathology.

### CCL3 Exacerbates Intestinal Damage in Experimental NEC

**Figure 2:**
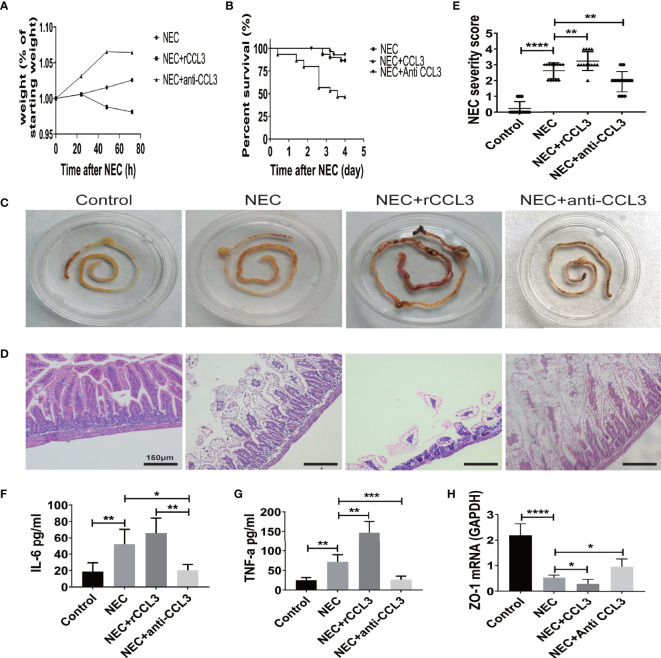
CCL3 exacerbated intestinal damage in the pathogenesis of NEC. **(A)** Weight changes of surviving pups in the group of NEC (n=9), NEC+rCCL3 (n=8), and NEC+anti-CCL3 (n=10). **(B)** After modeling, survival was observed in the NEC group, NEC+rCCL3 (50 ng/mL) group, and NEC+ anti-CCL3 (50 ng/mL) group. n=30 for each group. Comparison of survival curves was by Log-rank (Mantel-Cox) test. **(C)** Macroscopic view of mouse intestinal tissue from Control, NEC, NEC+rCCL3, NEC+anti-CCL3 groups. **(D)** Representative images of H&E-stained sections from Control, NEC, NEC+rCCL3, NEC+anti-CCL3 groups (Scale Bar =150 μm) **(E)** Histopathological scores in Control, NEC, and NEC+rCCL3, NEC+anti-CCL3 groups, as shown in **(D)**. Data are expressed as mean ± SD. **p < 0.01, ****p < 0.0001 when compared between groups (Mann-Whitney U test). **(F-G)** IL-6, TNF-α expression in the intestinal lysates of Control, NEC, NEC+rCCL3, NEC+anti-CCL3 groups (n-5-8). Data are expressed as mean ± SD. *p < 0.05, **p < 0.01, ***p < 0.001 when compared between groups (Mann-Whitney U test). **(H)** ZO-1 mRNA expression in the intestinal tissue of Control, NEC, NEC+rCCL3, NEC+anti-CCL3 groups were assessed by RT-PCR (n =4-6/group). Data are expressed as mean ± SD. *p < 0.05, ****p < 0.0001 when compared between groups (Mann-Whitney U test).

To characterize the role of CCL3 in the pathological process of NEC, mice in the intervention group were intraperitoneally injected with the rCCL3 or anti-CCL3 before modelling and maintained at an appropriate maintenance dose after modelling, Mice in the control group were intraperitoneally injected with PBS. The survival and intestinal injury of the mice were then assessed, and the results showed that, compared with that of the NEC+PBS control group, the NEC+rCCL3 group had slower or decreased weight gain (**Figure 2A**), increased mortality (**Figure 2B**), and more severe gross intestinal flatulence and bleeding (**Figure 2C**), whereas anti-CCL3 significantly reversed the rCCL3-mediated severe intestinal injury and death (**Figures 2A–C**). Consistent with the above results, pathological results showed that mice in the NEC+rCCL3 group had more severe intestinal tissue damage than those in the NEC+PBS control group, manifested as necrotizing changes and shedding of intestinal epithelial cells and the resulting thinning of the intestinal mucosal layer. In contrast, anti-CCL3 neutralized rCCL3-mediated severe intestinal epithelial damage (**Figures 2D, E**).

Previous studies have indicated that overwhelming inflammation is another important characteristic of NEC and is responsible for intestinal injury during NEC. Therefore, we investigated whether intestinal damage mediated by CCL3 was associated with modulation of the inflammatory response. ELISA was performed, and the results showed that IL-6 and TNF-α levels were significantly elevated in NEC mice compared to those in these control group, which were ever higher with exogenous rCCL3 treatment. Consistent with the observations, anti-CCL3 treatment reduced the release of these inflammatory factors (**Figures 2F, G**). In addition, we found that CCL3 inhibited the expression of the tight junction protein ZO-1, the deficiency of which is associated with intestinal epithelial cell shedding (**Figure 2H**). Taken together, these results indicated that CCL3 aggravates intestinal injury in NEC mice.

### CCL3 Aggravates Intestinal Epithelial Cell Apoptosis of NEC

**Figure 3:**
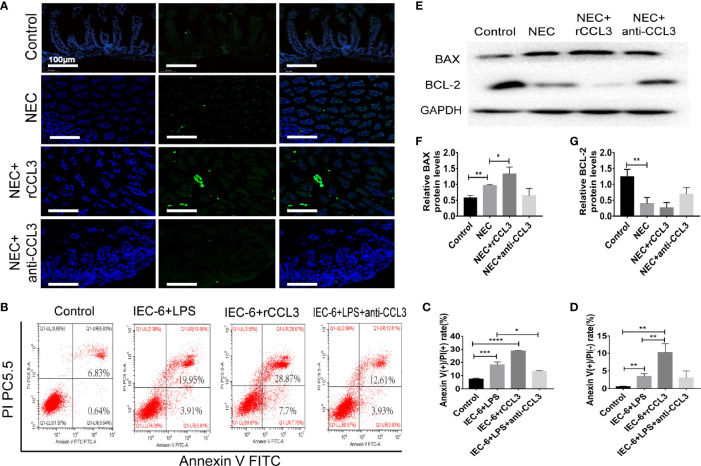
CCL3 aggravates the apoptosis of intestinal epithelial cells. **(A)** Representative images of TUNEL (FITC) stained sections from control, NEC, NEC+rCCL3, NEC+anti-CCL3 groups (Scale bar =100 μm). **(B-D)** IEC-6 cells were pretreated with rCCL3 (50 ng/mL), anti-CCL3 (50ng/ml) for 24h and exposed to 100μg/ml LPS for 4 h. Flow cytometry (FCM) was performed to evaluate the effects of rCCL3 and anti-CCL3 on the apoptosis of the IEC-6 cells. Data are expressed as mean ± SD. *p < 0.05, **p < 0.01, ***p < 0.001, ****p < 0.0001 when compared between groups. (Mann-Whitney U test) **(E-G)** The protein level of BAX and BCL-2 were assessed by Western blotting and the intensity was analyzed (n =4-6/group). Three independent experiments were performed. Data are expressed as mean ± SD. *p < 0.05, **p < 0.01 versus control or intervention groups (Mann-Whitney U test).

Intestinal injury in NEC is closely related to apoptosis, therefore, we hypothesised that CCL3-mediated shedding of intestinal epithelial cells may be related to apoptosis. To test this, paraffin sections of small intestinal tissue were prepared, and IF assays were used to detect apoptosis in the intestinal tissue of NEC mice. TUNEL (FITC fluorescence) results showed significantly increased intestinal tissue cell apoptosis in NEC model mice compared with that of the control group, and more severe apoptosis was observed in rCCL3-treated mice than in the NEC group. Intestinal tissue cell apoptosis in the anti-CCL3 group was significantly lower than that in both the NEC and NEC+rCCL3 groups (**Figure 3A**). To further verify the effects of CCL3 on intestinal epithelial cell apoptosis, a cell inflammatory model was constructed using IEC-6 cells (a small intestinal epithelial cell line) under LPS stimulation. In accordance with the results of IF staining, FCM showed that rCCL3 pre-treatment significantly enhanced LPS-induced IEC-6 cell apoptosis compared to those without rCCL3 treatment, while anti-CCL3 pre-treatment significantly decreased LPS-induced apoptosis of IEC-6 cells (**Figures 3B–D**). To determine how CCL3 induces apoptosis, the expression of the apoptosis-related factors BAX and BCL-2 was analysed in the intestinal tissues of NEC mice. We found that, with rCCL3 pre-treatment, the expression of the pro-apoptotic factor BAX was upregulated, while that of the anti-apoptotic factor BCL-2 in intestinal tissues was downregulated compared to that in the control group. Notably, anti-CCL3 treatment significantly counteracted the upregulation and downregulation patterns of these two proteins, even to levels similar to those of the control and NEC groups (**Figures 3E–G**). Together, these results support the notion that CCL3 promotes intestinal damage during NEC development by inducing intestinal epithelial cell apoptosis.

### CCL3 Promotes Apoptotic Intestinal Injury During NEC in a CCR4-Dependent Manner

**Figure 4:**
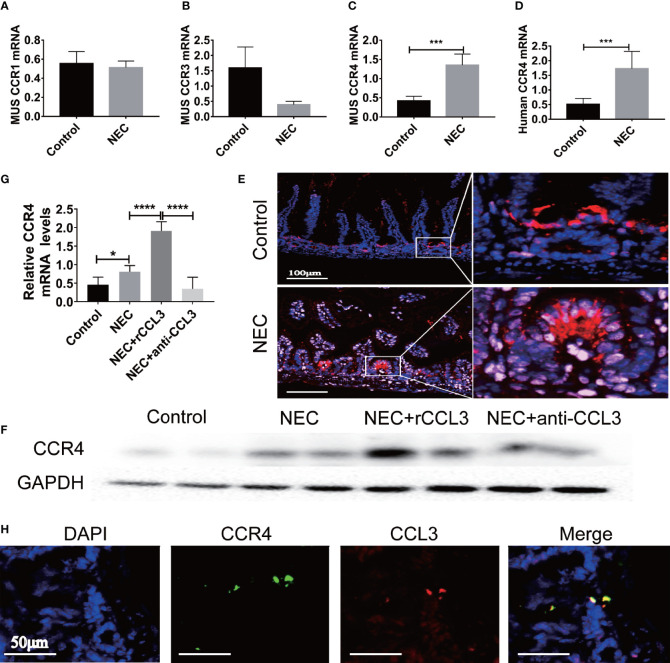
CCL3 mediates intestinal injury by binding to CCR4 during NEC. **(A-C)** The mRNA expression of CCR1, CCR3, CCR4 in mice with NEC were determined by RT-PCR (n =4-6/group). ***p < 0.001 when compared with control group. (MannWhitney U test) **(D)** CCR4 mRNA expression in patients with NEC were detected by RT-PCR (n =4-6/group). ***p < 0.001 when compared with control group. (MannWhitney U test) **(E)** Representative Fluorescence micrographs from sections of the terminal ileum in mouse with or without NEC, immunostained for CCR4 (CY5-Tyramide conjugated anti-CCR4) as indicated (Scale bar=100 μm). **(F, G)** CCR4 in intestinal was determined by WB and the intensity was analyzed, compared with control, NEC, NEC+rCCL3 and NEC+anti-CCL3 groups. *p < 0.05, ****p < 0.0001 when compared between groups. (Mann-Whitney U test) **(H)** Immunofluorescence co-location assay was performed, incubation with anti-CCL3 (CY3-Tyramide), anti-CCR4(FITC), anti-E-Cad (a calcium-dependent transmembrane protein distributed in various epithelial cells) and DAPI as indicated (Scale bars: 50 μm).

After observing the critical role of CCL3 in NEC-related intestinal epithelial cell apoptosis, we aimed to determine the potential mechanisms involved in the pro-apoptotic functions of CCL3. Previous studies have shown that CCL3 may bind to different receptors (CCR4, CCR1, and CCR3) and play various physiological and pathological roles in different diseases. To identify the target receptors that bind to CCL3 in NEC, the expression levels of these receptors in intestinal tissues were detected by RT-PCR, and no obvious difference was identified in the expression levels of CCR1 in the intestinal tissues of NEC mice compared to those of the control mice (**Figure 4A**). Notably, CCR3 expression levels decreased (**Figure 4B**), whereas those of CCR4 increased in NEC mice (**Figure 4C**), which was concomitant with the observed increased CCL3 expression. Elevated CCR4 expression in the intestinal tissue samples of clinical NEC patients was also found in the intestinal tissue of NEC mice (**Figure 4D**), which was further verified by IF staining (**Figure 4E**) and western blot analysis (**Figure 4F**). Furthermore, anti-CCL3 treatment significantly reduced NEC-induced CCR4 expression (**Figures 4F, G**). Notably, IF staining showed that co-localization of CCL3 and CCR4 in the intestine further provided an intuitive result of the direct binding of CCL3 to CCR4 (**Figure 4H**), suggesting that CCR4 may participate in CCL3-mediated NEC pathogenesis.

**Figure 5:**
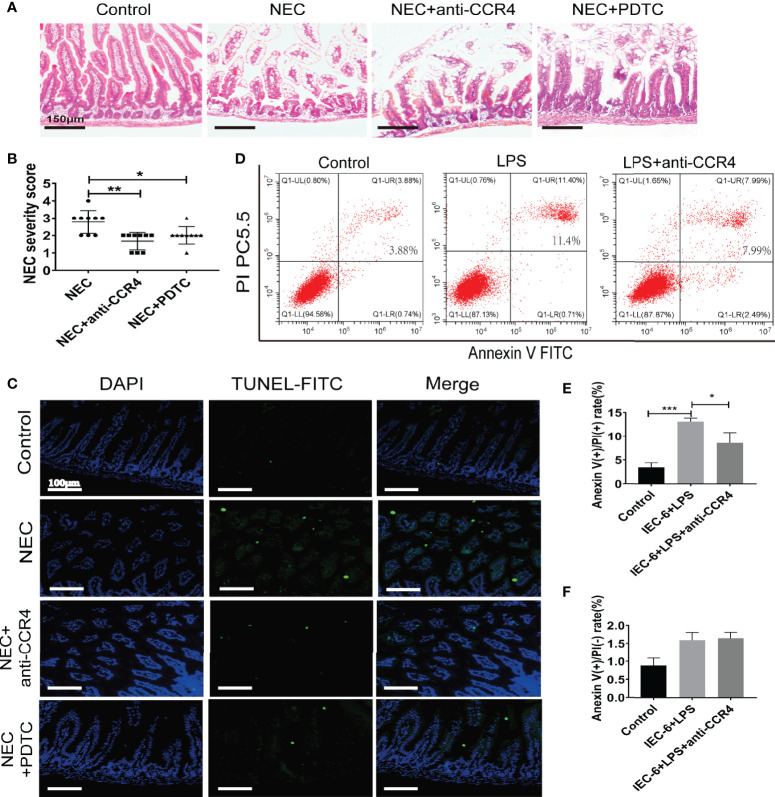
CCL3 mediates intestinal injury by binding to CCR4 during NEC. **(A)** Representative images of H&E stain from control, NEC, NEC+anti-CCR4 and NEC+PDTC groups (Scale bar =150 μm). **(B)** Histopathological scores in Control, NEC, NEC+anti-CCR4 and NEC+PDTC groups, as shown in **(A)**. *p < 0.05, **p < 0.01 when compared between groups. (Mann-Whitney U test) **(C)** Representative images of TUNEL (FITC) stained sections from control, NEC, NEC+anti-CCR4 and NEC+PDTC groups (Scale bar =100 μm). **(D-F)** FCM was performed to evaluate the effects of anti-CCR4 on the apoptosis of IEC-6 cells. Anexin (+) PI (-) represents early apoptosis and Anexin (+) PI (+) represents late apoptosis. Data are expressed as mean ± SD.*p < 0.05 ***p < 0.001 versus control or treatment groups. (Mann-Whitney U test).

To verify this, CCR4 was blocked using a CCR4 antibody (anti-CCR4) in NEC mice. Importantly, CCR4 blockade resulted in reduced tissue damage accompanied by reduced NEC histopathological scores (**Figures 5A, B**). Notably, anti-CCR4 treatment significantly attenuated NEC-induced intestinal tissue apoptosis (**Figure 5C**) and LPS-induced IEC-6 cell apoptosis (**Figures 5D–F**). Taken together, these results suggest that CCL3-CCR4 is an important cause of tissue damage and intestinal epithelial apoptosis in NEC.

### The CCL3-CCR4 Axis Mediates Apoptosis of Epithelial Cells Involved in the Activation of the ERK1/2-NKκB-BAX/BCL2 Signalling Pathway

**Figure 6:**
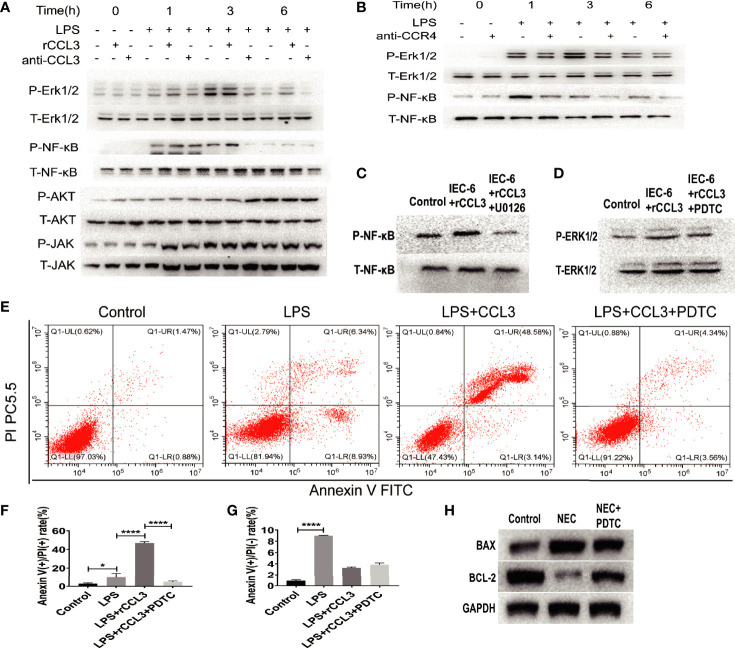
CCL3 activates apoptosis-related signaling pathways through the CCL3-CCR4-ERK1/2-NK-κB signal axis. **(A)** IEC-6 cells were treatment with LPS, LPS+rCCL3 or LPS+anti-CCL3, then the cells were harvest at different time (0,1,3,6h) for signal molecule phosphorylation detection by WB. **(B)** IEC-6 cells were treatment with LPS or LPS+anti-CCR4, then the cells were harvest at different time (0,1,3,6h) for signal molecule phosphorylation detection by WB. **(C)** IEC-6 cells were treated with PBS control, rCCL3, or rCCL3+U0126 (an inhibitor of ERK1/2), and the phosphorylation of NF-κB was detected by western blotting. **(D)** IEC-6 cells were treated with PBS control, rCCL3, or rCCL3+PDTC, and the phosphorylation of ERK1/2 was detected by western blotting. **(E-G)** FCM was performed to evaluate the effects of PDTC on the apoptosis of the LPS-induced IEC-6 cells. Anexin (+) PI (-) represents early apoptosis and Anexin (+) PI (+) represents late apoptosis. *p < 0.05, ****p < 0.0001 when compared between groups. (Mann-Whitney U test) **(H)** NEC modeling mice were treated with PBS control or PDTC, then BAX and BCL2 expression in intestinal tissue were detected by western blotting.

Despite evidence supporting the role of CCL3-CCR4 in intestinal tissue damage by inducing intestinal epithelial apoptosis, it remains unclear how CCL3-CCR4 causes intestinal epithelial cell apoptosis at the molecular level. Therefore, we assessed the apoptosis-related signalling pathways in NEC. IEC-6 cells were harvested for protein extraction after LPS stimulation, with or without rCCL3/anti-CCL3/antiCCR4 treatment, at different time points (0–6 h). Western blotting showed that rCCL3 pre-treatment significantly enhanced the phosphorylation levels of ERK1/2 and NF-κB in IEC-6 cells under LPS stimulation for 1–3 h, while both anti-CCL3 and anti-CCR4 pre-treatment blocked the enhanced phosphorylation levels of ERK1/2 and NF-κB by LPS and rCCL3 (**Figures 6A, B**, and densitometry analyses were shown in S Fig.1 and S Fig.2). No differences were observed in the phosphorylation levels of AKT and JAK in IEC-6 cells treated with or without rCCL3. Next, the sequential activation of NF-κB and ERK1/2 was determined. The phosphorylation of NF-κB in IEC-6 cells decreased after inhibition of ERK1/2 with U0126 (**Figure 6C**), while there was no significant change in the phosphorylation of ERK1/2 after inhibition of NF-κB with PDTC (**Figure 6D**), suggesting that ERK1/2 signalling is the upstream signalling molecule of NF-κB in the context of CCL3-induced apoptosis. Considering that NF-κB is an important intracellular nuclear transcription factor associated with apoptosis, the effects of the NF-κB pathway on apoptosis were verified. FCM analysis revealed that IEC-6 cell apoptosis was significantly reduced when NF-κB was inhibited (**Figures 6E–G**). More importantly, *in vivo* studies also showed that NF-κB inhibition significantly promoted the expression of the apoptosis inhibitor BCL-2 (**Figure 6H**) and alleviated NEC-induced severe intestinal tissue damage in mice (**Figure 5A**). Taken together, these findings uncover a previously unrecognized role of CCL3 in NEC-associated intestinal epithelial cell apoptosis in a CCL3-CCR4-ERK1/2-NF-κB-BAX/BCL2 axis-dependent manner. Our findings provide new strategies for the prevention and treatment of this devastating disease.

## Discussion

NEC is one of the most common and devastating gastrointestinal emergencies during the neonatal period. In the current study, we elucidated a previously unrevealed pathological mechanism of NEC development, in which CCL3 aggravates NEC-related intestinal damage through the CCL3-CCR4-ERK1/2-NK-κB-BAX/BCL2 signalling axis. This study provides a new treatment strategy for NEC. Clinical studies have indicated that the imbalance between pro-apoptosis and anti-apoptosis is a potential pathogenesis of NEC, and appropriately targeted blocking of apoptosis signals may be an effective strategy for NEC treatment (25). In this study, we used *in vivo* animal models and *in vitro* cell models to determine that CCL3 was highly expressed in intestinal lesions and directly induced intestinal epithelial cell apoptosis and the inflammatory response in NEC, which, to a large extent, directly promotes the occurrence and development of NEC. Further mechanistic studies revealed that this process is associated with the CCL3-CCR4-ERK1/2-NF-κB-BAX/BCL-2 signalling pathway, and blocking either CCL3, CCR4, or NF-κB with neutralizing antibodies or inhibitors can reverse CCL3-mediated intestinal epithelial apoptosis and NEC-related intestinal injury, which provides a new approach for immunotherapy of NEC.

There are several different functional receptors of CCL3, and by binding to them, CCL3 performs various biological functions in different diseases (26–29). Here, we illustrated that CCL3 mediates intestinal epithelial apoptosis in a CCR4-dependent manner. Our data showed that CCR4 and CCL3 were synchronously upregulated during NEC development, and immunofluorescence co-localization intuitively suggested their binding. Blocking CCL3 decreases CCR4 expression, and blockade of CCR4 also alleviates CCL3-mediated intestinal epithelial cell apoptosis. Previous studies have shown that CCR4, as a G-protein coupled receptor, plays an important role in immune regulation in various diseases (30–33). Our study revealed a novel mechanism for NEC-associated intestinal epithelial cell apoptosis based on the CCL3-CCR4 pathway.

ERK1/2 phosphorylation is closely related to apoptosis (34–36), and the downstream signalling of CCR is related to ERK1/2 phosphorylation (37). We demonstrated that phosphorylation of both ERK1/2 and NF-κB is responsible for rCCL3-induced intestinal epithelial cell apoptosis. Furthermore, NF-κB is one of the downstream pathways associated with ERK1/2 (38, 39), and the expression of apoptosis-related molecules BCL-2 and BAX is NF-κB-dependent (40, 41). It is simple to determine the relationship between ERK1/2 and NF-κB, and the ERK1/2-NF-κB pathway was determined to be unidirectionally activated. However, although our results and those of most researchers have demonstrated the activation of the ERK1/2/NF-κB pathway, other studies have identified the activation of NF-κB/ERK1/2. Different signaling pathways may be regulated by different intracellular signalling processes under different physiological and pathological conditions. However, whether CCL3-mediated inflammation is associated with phosphorylation of ERK1/2 and NF-κB remains unclear.

In this study, we focused on the effect of the CCL3-CCR4 axis on intestinal injury by inducing intestinal epithelial cell apoptosis. However, several other related questions remain unclear, such as how CCL3 is induced and from which cells. Previous studies have suggested that TLR4 is closely related to the occurrence and development of NEC, however, whether the production of CCL3 during NEC is related to TLR4 has not yet been verified. In addition, whether CCL3 affects NEC *via* chemotaxis of other immune cells requires further study. Although these questions were not taken as research objectives in this study, they are still worth discussing and exploring further.

In the current study, we provided direct evidence that CCL3 mediates NEC-related intestinal injury by exacerbating intestinal epithelial cell apoptosis through the CCL3-CCR4- ERK1/2-NF-κB-BAX/BCL-2 pathway, which helps us to further reveal the pathogenic mechanisms of NEC. It represents an important conceptual advance that CCL3 may be one of the key culprits of intestinal tissue damage in patients with NEC. Therefore, blocking CCL3 or CCR4 may be a novel and effective immunotherapy for NEC.

In conclusion, NEC is one of the most common and devastating gastrointestinal emergencies during the neonatal period. Our study elaborated a previously unrecognized role of the CCL3-CCR4 axis in the occurrence and development of NEC, which represents an important conceptual advance that CCL3 may be one of the key culprits of intestinal tissue damage in patients with NEC, and blocking CCL3, CCR4, or NF-κB may represent a novel effective immunotherapy for NEC.

## Data Availability Statement

The original contributions presented in the study are included in the article/**Supplementary Material**. Further inquiries can be directed to the corresponding authors.

## Ethics Statement 

The studies involving human participants were reviewed and approved by the Ethics Committee of Children’s Hospital of Chongqing Medical University. Written informed consent to participate in this study was provided by the participants’ legal guardian/next of kin. The animal study was reviewed and approved by the Ethics Committee of Chongqing Medical University.

## Author Contributions

ZS and DC conceived and designed the experiments. XY, ZX, YL, and HL performed the experiments. WL, XZ, YY, and JC collected and analyzed the data. ZS, DC, PX, and. XY design the study and writing of the manuscript. All authors had reviewed and approved the final submitted and published versions.

## Funding

The study was supported, in part, by the National Natural Science Foundation of China (Grant Number: 81801956 to ZS), Chongqing Municipal Health Commission (Grant Number: 2020MSXM050 to DC), Distinguished Young Scholars of the Children’s Hospital of Chongqing Medical University (ZS).

## Conflict of Interest

The authors declare that the research was conducted in the absence of any commercial or financial relationships that could be construed as a potential conflict of interest.

## Publisher’s Note

All claims expressed in this article are solely those of the authors and do not necessarily represent those of their affiliated organizations, or those of the publisher, the editors and the reviewers. Any product that may be evaluated in this article, or claim that may be made by its manufacturer, is not guaranteed or endorsed by the publisher.
