# Supplementary material for: Novel Therapeutic Targeting of CCL3-CCR4 Axis Mediated Apoptotic Intesitnal Injury in Necrotizing Enterocolitis
Source: Front Immunol. 2022 Apr 22;13:859398. doi: 10.3389/fimmu.2022.859398 (PMC9073010; doi:10.3389/fimmu.2022.859398)
Supplement: Supplementary file 1 [file DataSheet_1.docx]

Table 1. Gene-Specific Oligonucleotide Primers Used for Q-PCR

| Gene | Serial number | Primer sequence | products (bp) |
| --- | --- | --- | --- |
| mCCR4 | NM_009916 | F: 5′-TGCACCAAGGAAGGTATCAAGG-3′  R: 5′ -GTACACGTCCGTCATGGACTT-3′ | 147 |
| mCCR1 | NM_009912 | F: 5′-CTCATGCAGCATAGGAGGCTT-3′  R: 5′ -ACATGGCATCACCAAAAATCCA-3′ | 142 |
| mCCR3 | NM_009914 | F: 5′-TCGAGCCCGAACTGTGACT-3′  R: 5′ -CCTCTGGATAGCGAGGACTG-3′ | 143 |
| mZO-1 | NM_001163574 | F: 5′-GCTTTAGCGAACAGAAGGAGC-3′  R: 5′ -TTCATTTTTCCGAGACTTCACCA-3′ | 156 |
| hCCR4 | NM_005508 | F: 5′-AGAAGGCATCAAGGCATTTGG-3′  R: 5′ -ACACATCAGTCATGGACCTGAG-3′ | 137 |

Primers were designed from the published sequences in the GenBank database under the indicated accession numbers. F: forward primer; R: reverse primer; m: mouse; h: human.

Densitometry analyses and statistics for WB figures.

b. BAX (Fig.3F)

**
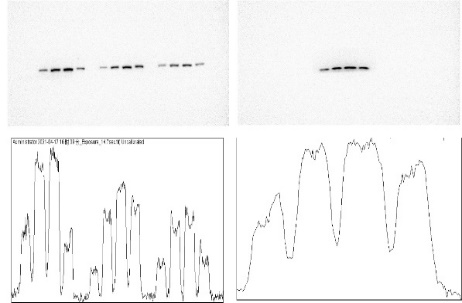

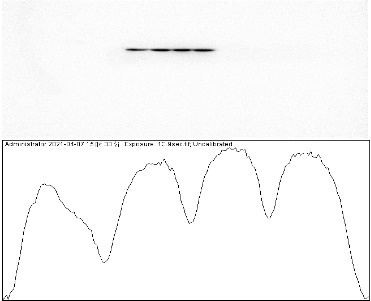

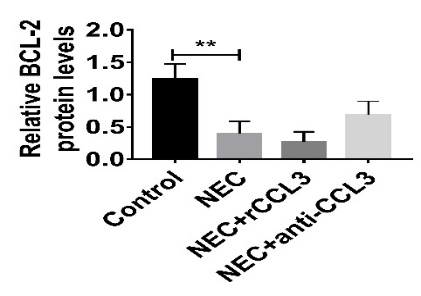
**

a. BCL2 (Fig.3G)

**
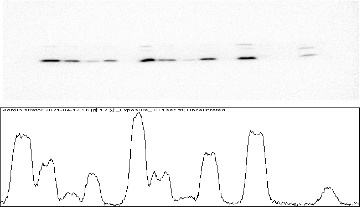

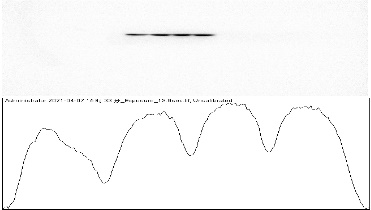

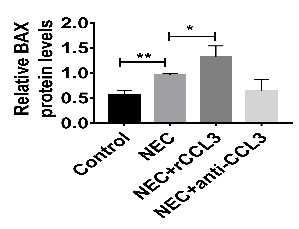
**

ERK1/2 (S Fig.1)

**
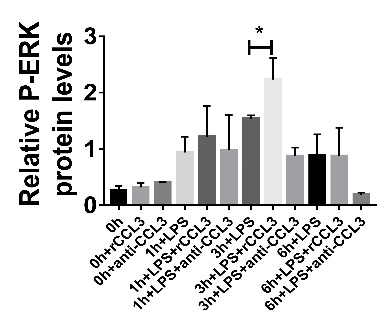

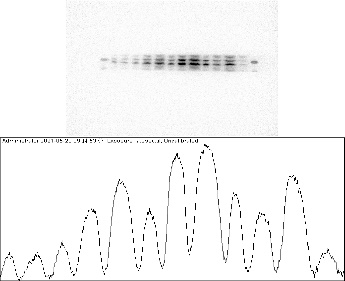

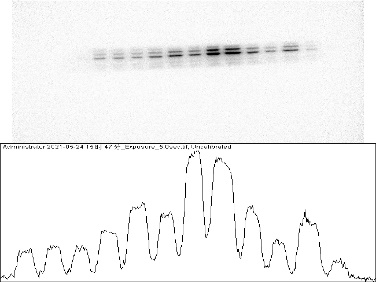

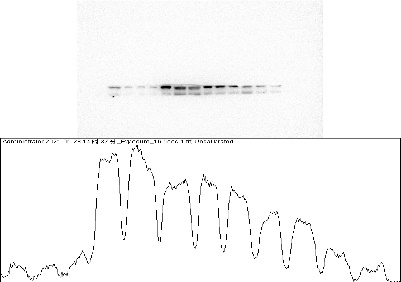

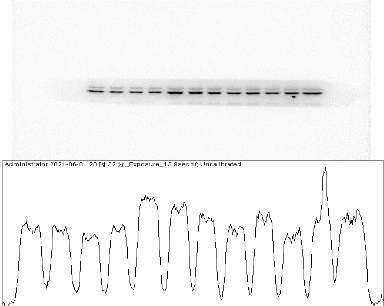
**

d. NF-κB (S Fig.2)

**
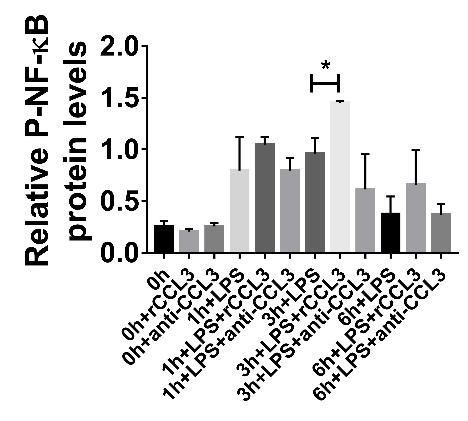

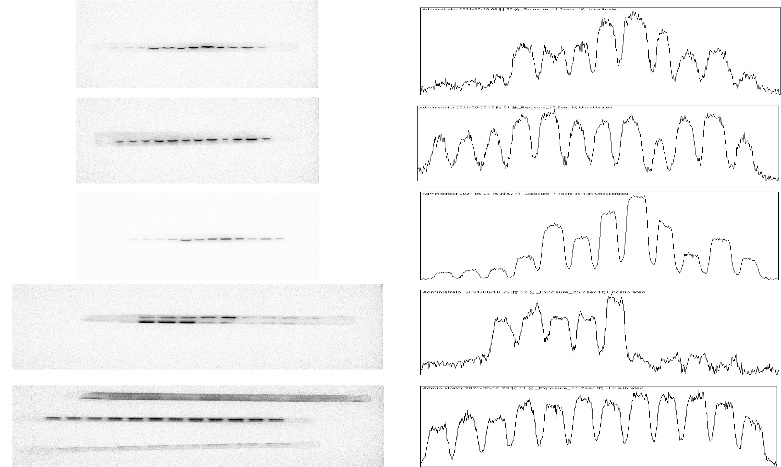
**
